# Shear mechanical response and deformation failure of F-type socket joint in a rectangular pipe jacking tunnel under different geologic conditions

**DOI:** 10.1038/s41598-023-49517-z

**Published:** 2023-12-11

**Authors:** Youjun Xu, Zhengrong Zhao, Chao Zhang, Xu Zhang, Yuekui Pang

**Affiliations:** 1 School of Civil Engineering, Inner Mongolia University of Science and Technology, Baotou, 014010 China; 2 Academician Workstation of Mine Safety and Underground Engineering, Inner Mongolia University of Science and Technology, Baotou, 014010 China; 3 Engineering Research Center of Urban Underground Engineering at Universities of Inner Mongolia Autonomous Region, Inner Mongolia University of Science and Technology, Baotou, 014010 China

**Keywords:** Engineering, Civil engineering

## Abstract

The shear mechanical properties of F-type socket joints in rectangular pipe jacking tunnels are currently unknown. To investigate the shear mechanical response and deformation failure of the F-type socket joint in rectangular pipe jacking tunnels under different foundation coefficients, a laboratory joint test and numerical simulation method were used, considering the structural features of the joint. The results showed that the deformation process of a joint subjected to shear consists of four stages: gap closure, elastic growth, shear strengthening, and yield failure. The ultimate shear capacity of the joint increases by 25% to 34% for every 3 mm increase in the steel ring thickness. The chamfer yield damage area comprises approximately 15% of the steel ring. The joint concrete crack first appears at the top of the socket joint, and the concrete damage area accounts for about 40% of the whole pipe section. The failure characteristics of the joint are primarily manifested as drum and warp of the steel ring or cracking of the weld, and the concrete at the joint is crushed. In practical engineering, the weld should not be located at the chamfer. The steel ring at the chamfer needs to be locally strengthened, and the chamfer and the reinforcement at the top and bottom need to be increased to improve the bearing capacity of the concrete.

## Introduction

**Figure 1:**
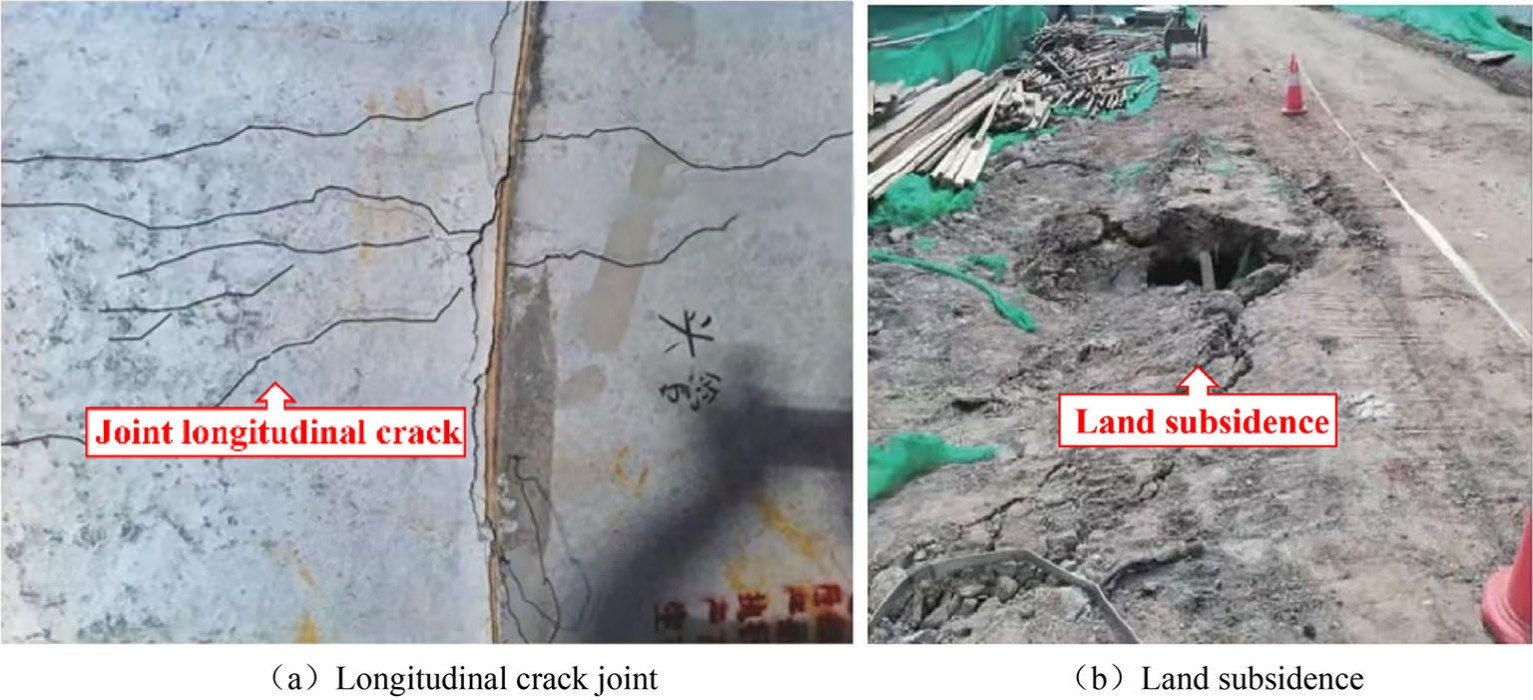
Rectangular pipe jacking operation failure.

As urban areas continue to grow, above-ground space is becoming increasingly scarce. Consequently, underground space development has become an essential component of urban development. The rectangular pipe method has played a crucial role in the construction of subway stations, underground utility tunnels, and other municipal projects, both domestically and internationally. The joint of the rectangular pipe jacking tunnel is a flexible joint formed by the connection of the steel ring, and the steel ring is an elastic material, which allows a certain deformation at the joint. Therefore, the stiffness of the pipe joint is much smaller than the stiffness of the pipe itself, which becomes the weakness of the rectangular pipe jacking tunnel. Excessive external stresses can easily result in joint opening or dislocation, leading to a loss of waterproofing or even damage. As depicted in Fig. 1, this poses a threat to structural safety and can even cause engineering calamities^1,2^.

Recent research on rectangular pipe jacking tunnels has been conducted by both domestic and foreign academics utilizing numerical simulation and actual projects. It concentrates primarily on the enhancement of rectangular pipe jacking construction technology, the impact of construction on land subsidence, the calculation of the jacking force and friction force, the optimization of anti-friction slurry ratio, and the stability excavation surface. Based on the Shasan Station project for Shenzhen Rail Transit Line 12, Wang et al.^3^ proposed a new type of high-performance joint for assembled subterranean structures. She et al.^4^ optimized critical construction parameters, including the soil bunker pressure ratio, grouting pressure ratio, and friction coefficient, for a rectangular pipe jacking project in Tianjin using numerical simulations. Using a rectangular pipe jacking project in Nanjing combined with numerical simulation, Zang et al.^5^ proposed a microexcavation construction method. Taking the Suzhou Chengbei Road Pipe Gallery Project as the background, Tang et al.^6^ compared the measured settlement value with the Peck formula and random medium theory, and proposed suggestions for predicting the ground settlement of large-section rectangular pipe jacking. Through three-dimensional numerical simulation, Ma et al.^7^ studied the distribution characteristics of ground subsidence troughs when the thickness of pipe jacking and the parallel distance of pipe jacking were changed. Based on the Mindlin solution and stochastic medium theory, Niu et al.^8^ proposed a superposition model of surface subsidence caused by friction, additional stress and soil loss in rectangular pipe jacking construction. With the Zhengzhou Zhongzhou Avenue pipe jacking project as the background, combined with numerical simulation, Xiao et al.^9^ proposed a total contact displacement control model considering grouting pressure to estimate the jacking force of large-section rectangular pipe jacking. Taking a project in Japan as an example, Ma et al.^10^ studied the ground response characteristics of rectangular pipe jacking with the same cross-sectional area but different aspect ratios by numerical simulation. Wen et al.^11^ proposed five classical analytical calculation models considering pipe-soil-mud interactions and obtained the prediction formula. The jacking force was estimated using a three-dimensional finite difference method. Liu et al.^12^ proposed two thixotropic slurry formulations for large-section rectangular pipe jacking in anhydrous sand. The microscopic slurry mechanism was studied using scanning electron microscopy. Li et al.^13^ used complex variable theory to analyse the mechanics of rectangular pipe jacking, and the complex variable solution of elastic half-plane rectangular pipe jacking was derived.

Existing research on the mechanical response and deformation failure of F-type socket joints in rectangular pipe jacking tunnels is lacking, according to the aforementioned research. Rectangular pipe jacking tunnels, shield tunnels, and integrated pipe galleries all have numerous joints and are longitudinally discontinuous cylinders, so, it is possible to borrow research ideas from existing research on force deformation at the joints of shield tunnels and integrated pipe galleries to further investigate rectangular pipe jacking tunnel joints. Zhu et al.^14^ performed prototype tests to analyse the deformation and stress of the concrete lining and joint of a quasirectangular shield tunnel and revealed the damage characteristics. Liu et al.^15,16^ used a prototype test to reveal the failure mechanism of the segment joint of a quasirectangular shield tunnel. Ding et al.^17^ studied the mechanical behaviour and deformation failure of shield tunnel segments and bolts under the combined action of axial force, bending moment and shear through a prototype test. Zhang et al.^18^ studied the mechanical properties and failure process of key segments of superlarge-diameter shield tunnels through prototype testing and revealed the mechanical properties and failure mechanism of key segments. Salemi^19^ used a direct shear test to study the mechanical behaviour of the longitudinal joint in the shield tunnel lining, and a clear relationship between the stiffness of the contact point and the normal stress of the contact position was proposed. Chen et al.^20^ used numerical simulation to study the failure mechanism, convergence deformation and structural stiffness of a shield tunnel lining. Dong et al.^21^ studied the relationship between crack development, failure mode of deformation joints, shear load and fault deformation of tunnel segments through experiments and numerical simulation.

In conclusion, domestic and foreign theoretical research on F-type socket joints in rectangular pipe jacking tunnels lags behind engineering practices. Lacking experimental verification and theoretical support, research on the mechanical response and deformation failure of F-type socket joints in rectangular pipe jacking tunnels is not systematic. For analysis of rectangular pipe jacking tunnel joints, only shield tunnel and underground utility tunnel joint research methodologies have been used in prior research. Compared with the joint between shield tunnel and underground utility tunnel, the F-type socket joint in rectangular pipe jacking tunnel has great differences in pipe joint shape, joint structure, section size, use function and waterproof grade. Rectangular pipe jacking is mostly flexible joint, while shield tunnel and underground utility tunnel are mostly rigid joint or semi-rigid joint, so the mechanism of joint action is also quite different.

Therefore, the context of this paper is the interchange underground passage project in Hohhot Hailiang Square, and the research object is an F-type socket joint in a rectangular pipe jacking tunnel. A combination of laboratory tests and numerical simulation methods were used to investigate the shear mechanical response and deformation failure of F-type socket joints in rectangular pipe jacking tunnels, filling a void in the current body of knowledge. The theoretical significance of the research findings for the design of joints in rectangular pipe jacking tunnels is substantial.

## Experimental design

### Pipe parameters

**Figure 2:**
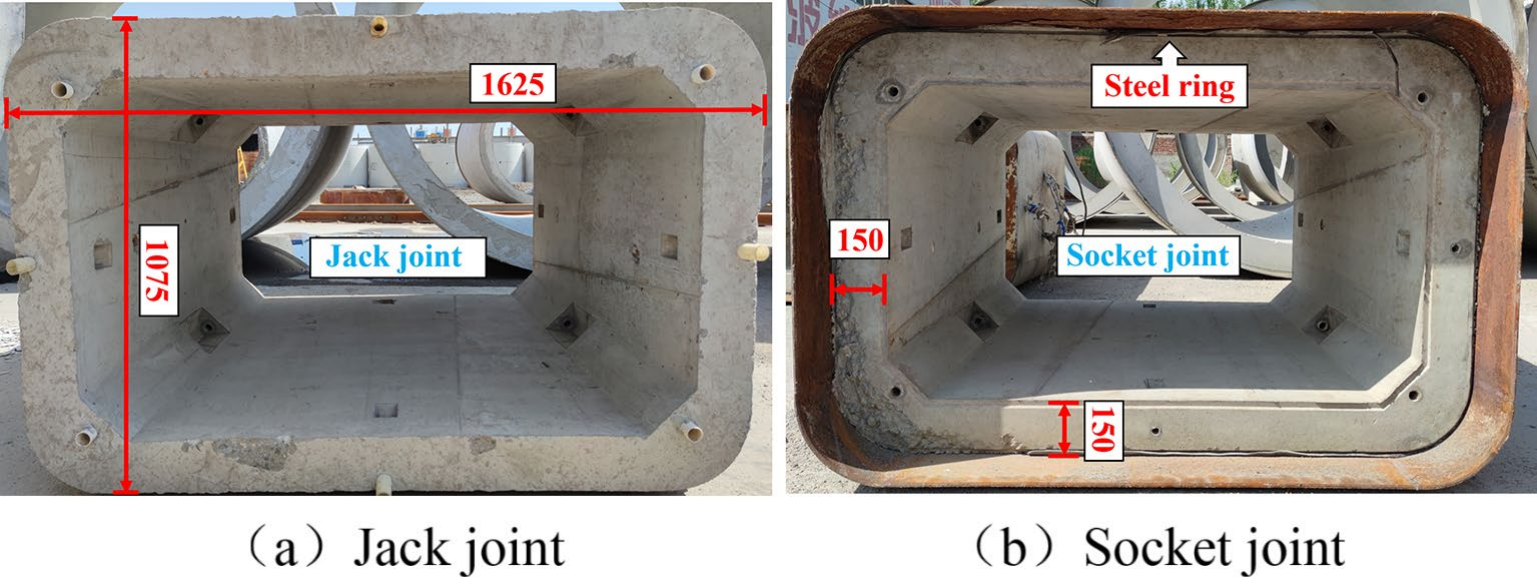
Joint construction.

**Figure 3:**
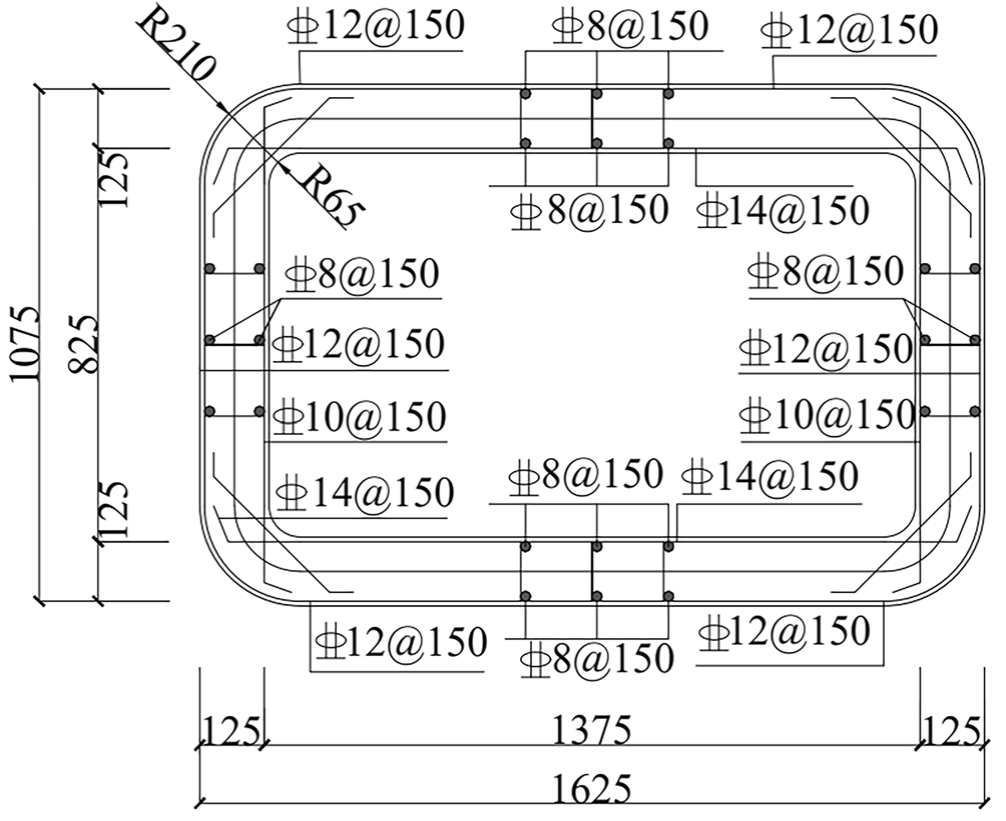
Reinforcement of the pipe section.

The pipe section is designed with a wall thickness of 150 mm and dimensions of 1500 mm by 1625 mm by 1075 mm. Steel rings, rubber rings, and steel cages make up the majority of the pipe section composition. Figure 2 depicts the joint's structure, while Fig. 3 depicts the reinforcement for the pipe section. The pipe section is composed of C50 concrete, and a 10 mm-thick Q235 steel ring is cast into the extremity of the pipe section. Table 1 displays the mechanical performance indicators for each material.

**Table 1:** Index of material mechanical properties.

Material	Concrete (C50)	Steel ring (Q235)	Rebar (HRB400)
Weight density (kN/m^3^)	25.0	78.5	78.5
Poisson's ratio	0.2	0.3	0.3
Young's modulus (MPa)	34,500	210,000	200,000
Yield strength (MPa)	–	235	400
Ultimate strength (MPa)	–	400	570
Standard compressive strength value (MPa)	38.0	–	–
Standard tensile strength value (MPa)	2.5	–	–

### Measurement system

**Figure 4:**
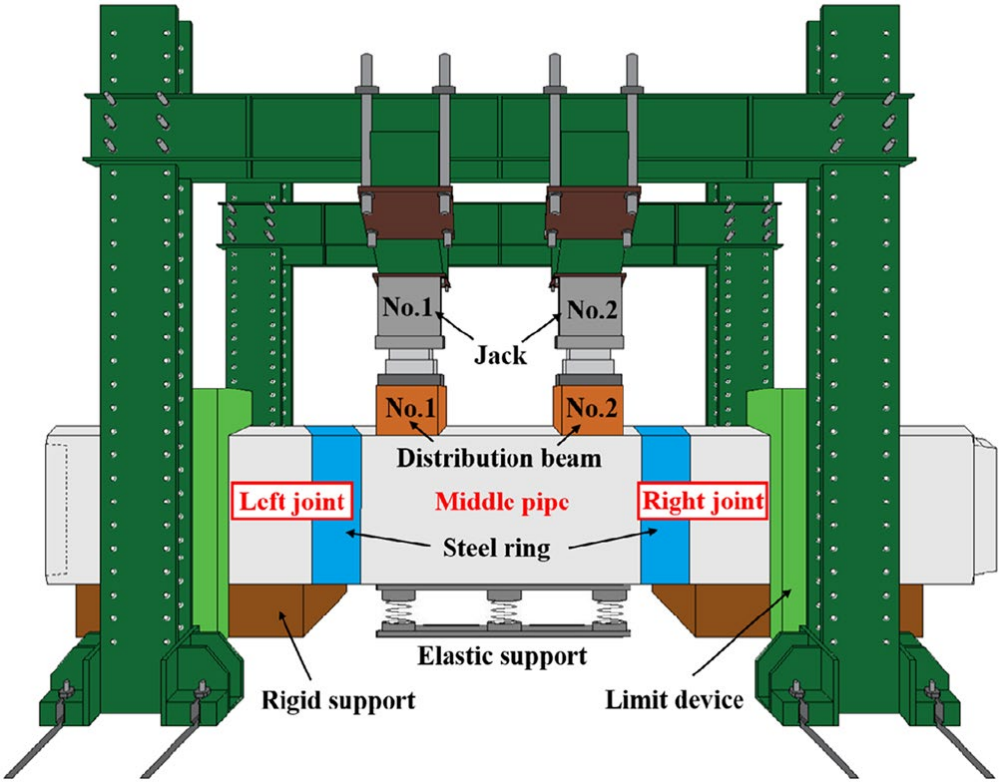
Test device.

The laboratory shear test for the F-type socket joint in rectangular pipe jacking consists of three pipe sections totalling 4.5 m in length. The primary components of the test system are jacks, computers, and a sensor data collection system. The two end pipe sections are fixed vertically with a limit device, and the jacks are positioned above the middle pipe section. The joint is loaded using the jack-distribution beam system to carry out the F-type socket joint shear test. The testing apparatus is depicted in Fig. 4.

**Figure 5:**
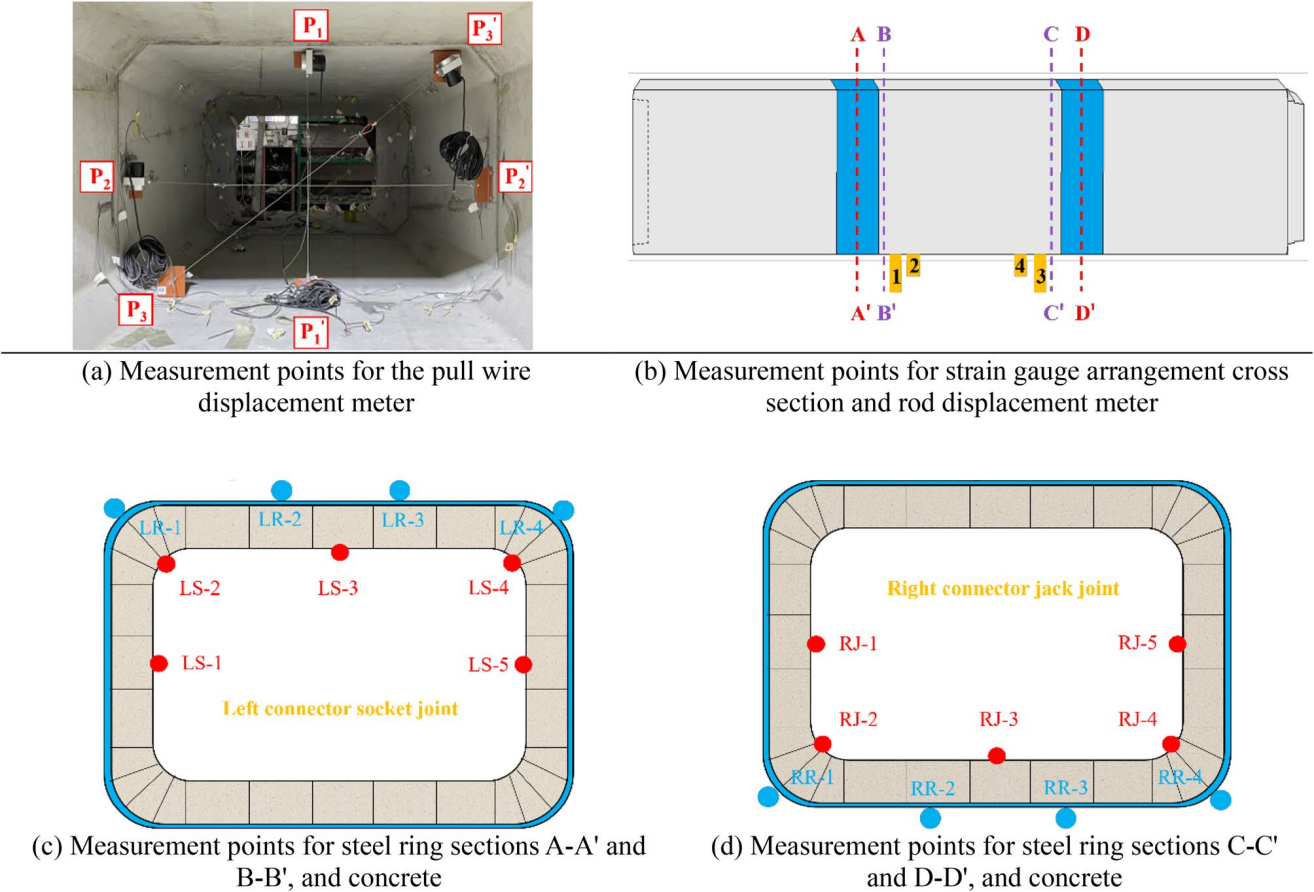
Measurement point arrangement.

The experiment measures the applied load at the joint, the displacement at multiple locations along the pipe section, and the strain values of the concrete, steel ring, and steel cages at the joint. As depicted in Fig. 5, strain gauges, rod displacement meters, and pull wire displacement meters are installed at the joint to examine the deformation of the concrete, steel ring, and steel cages during loading. The strain gauge has a resistance of 120 Ω, a sensitivity coefficient of 2.0 ± 0.01, and a strain limit of 20,000 µm/m. The measurement range of the pull wire displacement meter is 0–1250 mm, with a linearity of ± 0.5%. The deformation calculation formula is as follows:

$$ L = K\left( {U_{i} - U_{0} } \right), $$

where *L* represents the pull wire displacement meter displacement. *K* is the instrument calibration coefficient. *U*_*i*_ is the real-time pull wire displacement meter value. *U*_*0*_ is the initial pull wire displacement meter value.

### Test conditions

**Figure 6:**
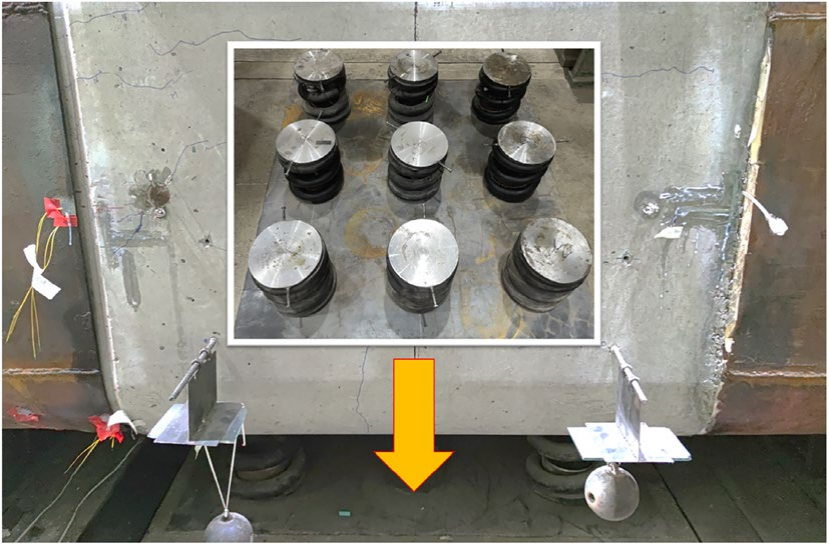
Spring arrangement.

In engineering practice, the effect of diverse foundation coefficients on joints is considered. Therefore, this experiment simulates the joint support by various soil layers using springs arranged uniformly at the lower portion of the middle pipe section, as depicted in Fig. 6. As shown in Table 2, the experiment is divided into three working conditions. The equivalent foundation stiffness coefficient can be calculated based on the principle that the foundation reaction force generated per unit displacement of the foundation is equal.

$$ n \cdot k = K_{v} \cdot S, $$

where* n* is the number of equivalent foundation springs, k is the spring stiffness used in the experiment, and its single spring stiffness was measured to be *k* = 1.783 kN/mm. *K*_*v*_ is the equivalent foundation stiffness coefficient, and *S* is the area of the pipe floor.

**Table 2:** Test conditions.

Working condition	Spring amount	Floor area (m^2^)	Equivalent foundation stiffness coefficient (× 10^3^ kN/m^3^)	Equivalent strata
Working condition 1 (WC-1)	9	1.43	10.16	Recent fill
Working condition 2 (WC-2)	6	1.43	6.77	Soft clay
Working condition 3 (WC-3)	4	1.43	4.52	Loose sand

### Loading system

**Figure 7:**
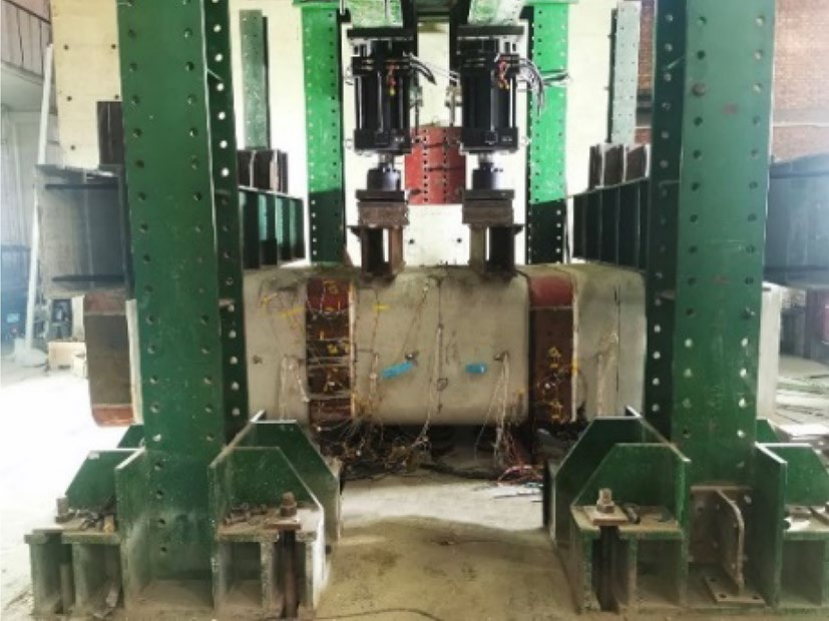
Loading device.

The experiment is loaded through regulated displacement^22^, using a jack for vertical displacement as a reference; the loading device is depicted in Fig. 7. The loading system consists of thirteen phases. The increase in displacement for stages 1–3 is 5 mm, for stages 4–7 it is 4 mm, for stages 8–10 it is 3 mm, and for stages 11–13 it is 2 mm. This is because the initial load and deformation of the joint are minor, but they grow over time. After each loading stage, a 5-min stabilization period is maintained to observe the pipe section's deformation and fracture formation. The experiment was terminated when the joint sustained substantial injury. During the experiment, the two jacks use synchronized and equal displacement increases to conduct the joint's shear test to prevent large vertical displacement differences in the middle pipe section from causing bending deformation of the joint.

## Shear test results of rectangular pipe jacking joints

### Macroscopic distortion of pipes

**Figure 8:**
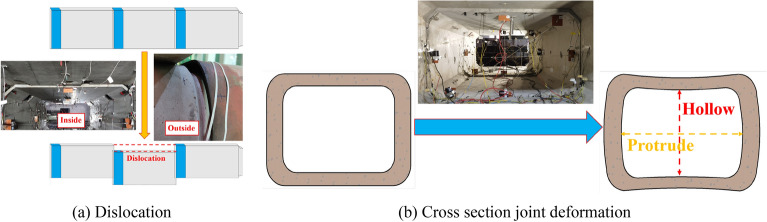
Macroscopic deformation of pipe sections.

Figure 8a is the dislocation deformation between the middle pipe section and the two end pipe section. Figure 8b is the deformation of the cross section of the middle pipe joint. In the experiment, the deformation of the cross section is small. In order to make the deformation look more obvious, the deformation is enlarged in the picture. When subjected to shear loads, the vertical displacement of the two end pipe sections is restricted, resulting in dislocation of the intermediate pipe section. Due to the restrictive effect of the steel ring and the compression of the joint from the load and foundation, uneven displacement and deformation occur at the joint, resulting in a tendency towards flattening in the cross-sectional direction of the joint, which causes the top and bottom of the joint to be concave towards the inside of the pipe, and the sidewalls of the pipe section to bulge outwards.

### Deformation of steel rings

**Figure 9:**
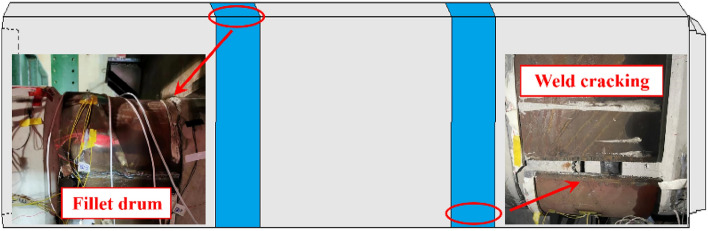
Deformation of steel rings.

Figure 9 depicts the deformation of the steel ring under the joint shearing state. The failure of the steel ring manifests itself primarily as drum at the chamfer and cracking at the weld. The reason for this is that when the joint produces shear deformation, the steel ring bears the majority of the shear. Throughout the process of resisting external loads, the concrete at the joint is continuously squeezed. Therefore, it is simple to produce stress concentration at the chamfer, resulting in drum of the steel ring in this area. In addition, the welding procedure weakens the edge of the material close to the weld, causing the steel ring at the weld's edge to tear under greater deformation. Therefore, the weld should not be located at the chamfer, and the steel ring at the chamfer should be locally strengthened.

### Joint concrete damage

**Figure 10:**
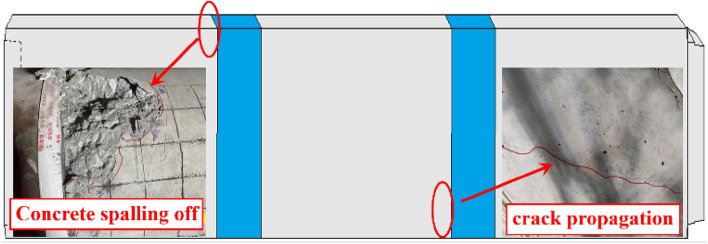
Concrete damage.

Figure 10 illustrates the failure of the concrete joint. As the shear load progressively increases, the contact pressure between the steel ring and the socket joint's concrete continues to increase, eventually causing the concrete near the contact surface of the steel ring to be crushed. Simultaneously, numerous diagonal cracks will appear at this location. As loading continues, the upper load and the reaction force of the pipe bottom springs continue to squeeze the pipe section, causing the pipe to undergo crushing failure, with partial diagonal cracks developing through each other. During the experiment, it is possible to observe the cracks at the joint continuously spreading and extending, along with the phenomenon of concrete spalling off. Therefore, it is necessary to increase the reinforcement at the chamfering position to enhance the bearing capacity of the concrete here.

## Analysis of joint shear test results

The left and right joints are included in the shear test. Because the jack joint is in the form of double steps and there is a steel ring in the socket joint, the stress and deformation of the two joints are different. A comparative analysis of the two joints was conducted so that the test's conclusion is more comprehensive.

### Joint shear

**Figure 11:**
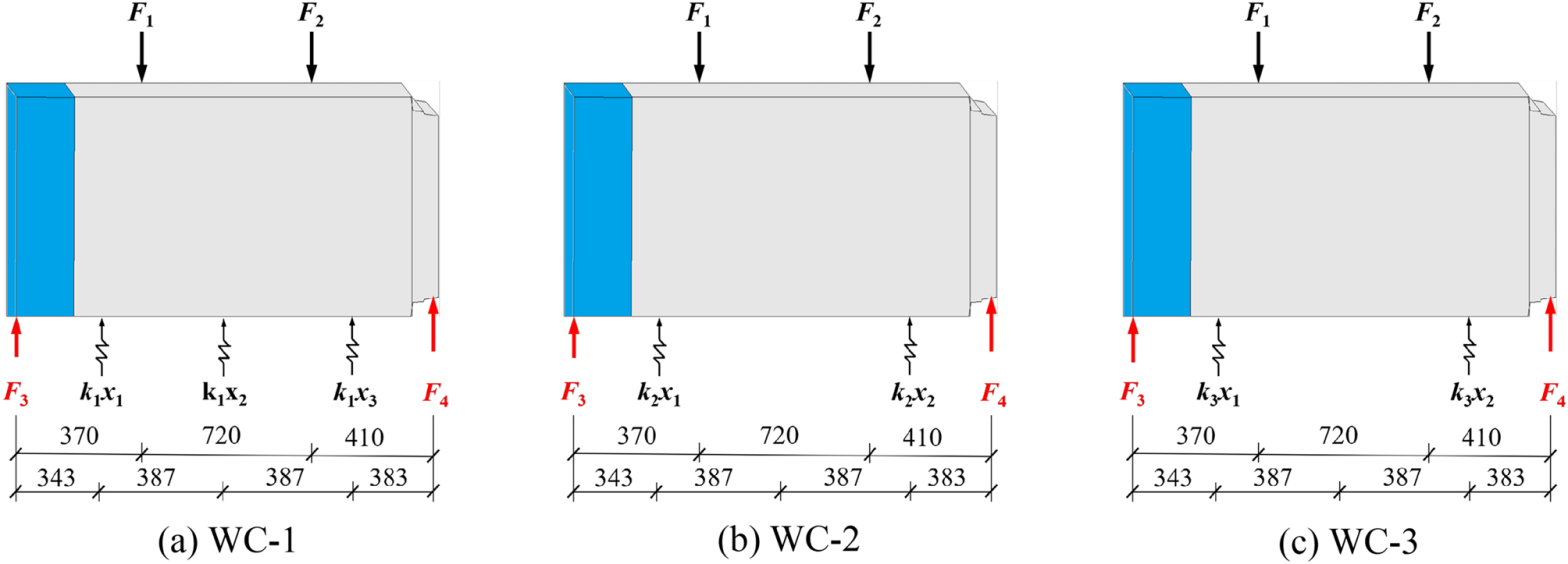
Force schematic diagram of pipe sections.

**Figure 12:**
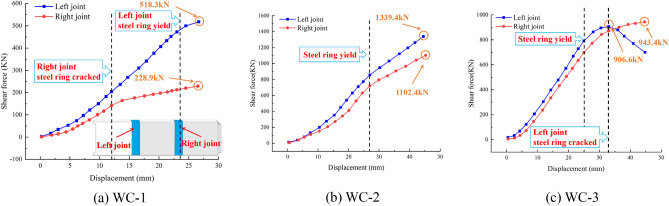
Shear changes of joints.

To investigate the variation in the joint's shear under different foundation coefficients, Fig. 11 depicts a schematic diagram of the force on the middle pipe section under each working condition, from which the shear *F*_3_ and *F*_4_ of the joint can be derived using the force balance Ʃ*F*_y_ = 0 and Ʃ*M* = 0. Here, *F*_1_ and *F*_2_ are the upper jack loads, *K*_i_*x*_j_ (i = j = 1,2) are the equivalent foundation spring reactions, *k*_1_ = *k*_2_ = 5.202 kN/mm, and *k*_3_ = 3.468 kN/mm. Figure 12 depicts the variation in the left and right joint shear under various conditions.

Under recent fill and considering the weakening effect of the joint, the weld of the right joint's steel ring cracked prematurely when loaded to 12 mm, resulting in an increase in the shear difference between the two joints when the loading displacement exceeded 12 mm. As the cracking area of the steel ring expanded, the shear strength progressively decreased, and a portion of the shear was borne by the bottom springs, resulting in the left joint's steel ring yielding earlier when loaded to 23 mm. Finally, the shear force of the left joint is 518.3 kN, while the shear force of the right joint is 228.9 kN. In subsequent working conditions, the steel ring's weld was strengthened.

Due to the different structure of the jack joint and the socket joint, when the loading displacement reached 10 mm, the shear trend of the left joint was greater than that of the right joint. When loaded to a distance of 27 mm, the shear trend of both joints slowed as the loading displacement increased. This indicates that the steel ring entered the yield stage at this time, and it was observed that the steel ring of both joints began to warp during the experiment. Finally, the shear force of the left joint is 518.3 kN, while the shear force of the right joint is 228.9 kN.

Under loose sand, when both joints were loaded to 25 mm, the increase in shear slowed as the loading displacement increased, and the steel ring of both joints began to warp, indicating that the steel ring began to yield and enter the strengthening stage. When the load reaches 33 mm, the shear force of the left joint reaches a maximum value of 906.6 kN, and then the shear force of the left joint begins to decrease. At this time, the weld seam of the left joint steel sleeve cracks, resulting in the release of the stress of the steel ring, and part of the shear force is transferred to the spring at the bottom of the pipe section. Finally, the shear force of the right joint reaches 943.4 kN.

In general, the shear rises slowly at the outset of loading. With an increase in loading displacement, the shear of both joints will increase before the steel ring reaches the yield point. When the steel ring enters the yield stage, the joint's shear growth rate will decelerate. Continued loading until the steel ring cracks will result in the steel ring's shear being lost and reduced. In addition, as the foundation coefficient weakens, the shear capacity at the joint will diminish.

### Joint dislocation

**Figure 13:**
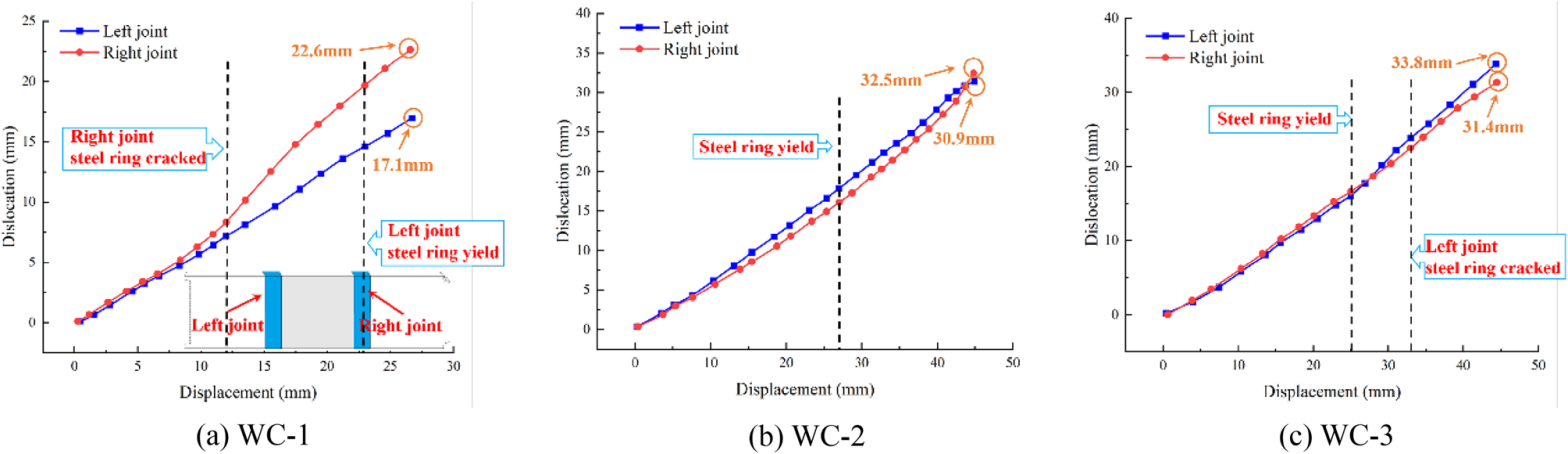
Dislocation changes of joints.

Figure 13 depicts the joint's displacements under each working condition to examine the variation of joint's dislocation under various foundation coefficients.

Before the loading displacement reaches 12 mm under recent fill, the change trend of the disparity of the left and right joints is identical, and their increase rates remain constant. However, when the loading reaches 12 mm, the steel ring of the right joint begins to crack, and the cracking area gradually expands with the loading process, resulting in decreased restraint and increased deformation tendency of the steel ring of the right joint, which causes the right joint's dislocation to increase at a faster rate than the left joint's. When the displacement reaches 23 mm, the yield of the steel ring of the left joint increases the rate of increase of the left joint's stagger. Finally, the difference between the dislocations of the two joints reaches 5.5 mm.

Under soft clay, when the loading displacement is less than 11 mm, the two joint dislocations are nearly identical. Nevertheless, when the loading displacement is between 11 and 27 mm, the increase rate of the left joint's dislocation begins to exceed that of the right joint due to the differences between the two joints. When the loading displacement reaches 27 mm, the rate of increase in the right joint's dislocation begins to surpass that of the left joint. This is because the steel ring of both joints enters the yield stage, and the degree of deformation of the steel ring in the right joint is greater than that of the left joint. Finally, the dislocation of the left joint is 30.9 mm, and the dislocation of the right joint is 32.5 mm.

Under loose sand, when the loading displacement is less than 25 mm, the two joint dislocations are essentially equal. When the loading displacement reaches 25 mm, however, the steel ring of both joints warps and enters the yield stage. As the loading displacement increases, the increase rate of the two joint dislocations also increases. When the loading displacement reaches 33 mm, the steel ring of the left joint cracks, accelerating the rate of increase of the left joint's dislocation. Finally, the dislocation of the left joint is 33.8 mm, and the dislocation of the right joint is 31.4 mm.

In summary, the dislocation of the two joints increases as the loading displacement increases, and the dislocation is less than the loading displacement, indicating that deformation occurs in the pipe's cross section. When the steel ring enters the yield stage under each working condition, the increase rate of the joints' dislocation will accelerate. When the loading continues until the steel ring cracks, the rate of dislocation of the joints will increase further. In addition, as the foundation coefficient weakens, the disparity between the two joints' dislocations will appear earlier, and the dislocation of both joints will eventually increase. When the girth weld of the steel sleeve is not cracked, the difference between the two joint dislocations is less than 2.5 mm.

### Cross-sectional deformation of joints

**Figure 14:**
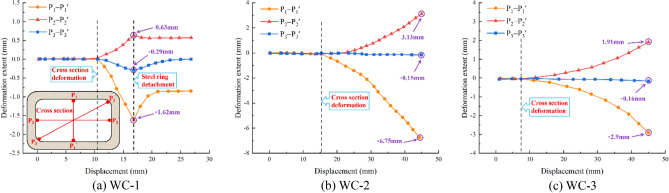
Change amount of pull wire displacement meter.

Figure 14 depicts the variations in displacement measured by pull wire displacement meters at the joint to research the cross-section changes of the joint under various foundation coefficients. Based on the changes in displacement measured by the pull wire displacement meter, it is possible to ascertain the deformation of the joint's cross section.

Under recent fill, when the displacement was within 10.5 mm, none of the three pull wire displacement meters exhibited significant changes, indicating that the joint's cross-sectional area did not endure significant deformation at this stage. P_1_-P_1_' and P_3_-P_3_' underwent compression deformation when the loading displacement was between 10.5 mm and 16.8 mm, while P_2_-P_2_' underwent tensile deformation. P1-P1' reached the maximum deformation of 1.62 mm, P2-P2' reached the maximum deformation of 0.63 mm, P3-P3' reached the maximum deformation of 0.29 mm. Due to the large crack in the chamfered steel ring, when the loading displacement exceeded 16.8 mm, the steel ring and the pipe section became detached, weakening the restraining effect on the joint and causing the joint to begin recovering its original shape.

Under soft clay, when the displacement was within 15.3 mm, there was no obvious deformation of the joint's cross-sectional area. When the loading displacement exceeded 15.3 mm, P_1_-P_1_' continued to experience compression deformation, P_2_-P_2_' continued to experience tensile deformation, and P_3_-P_3_' experienced minimal deformation throughout the entire loading process. The maximum deformation of P1-P1' is 6.75 mm, the maximum deformation of P2-P2' is 3.13 mm, and the maximum deformation of P3-P3' is 6.75 mm.

Under loose sand, when the displacement was within 7.4 mm, there was no obvious deformation of the joint's cross-sectional area. When the loading displacement exceeded 7.4 mm, P_1_-P_1_' continued to experience compression deformation, P_2_-P_2_' continued to experience tensile deformation, and P_3_-P_3_' experienced minimal deformation throughout the entire loading process. The maximum deformation of P1-P1' is 2.9 mm, the maximum deformation of P2-P2' is 1.91 mm, and the maximum deformation of P3-P3' is 0.16 mm.

Overall, P_1_-P_1_' exhibited compression deformation, P_2_-P_2_' exhibited tensile deformation, and P_3_-P_3_' exhibited minimal deformation as the upper load increased progressively. During shear loading, there was a tendency for the pipe section's cross section to flatten, and the deformation in the pipe section's diagonal direction was relatively small due to the limiting influence of the steel ring. As the foundation coefficient decreased, the cross section deformed more rapidly, the deformation rate decreased, and the ultimate amount of deformation decreased.

### Steel ring strain

**Figure 15:**
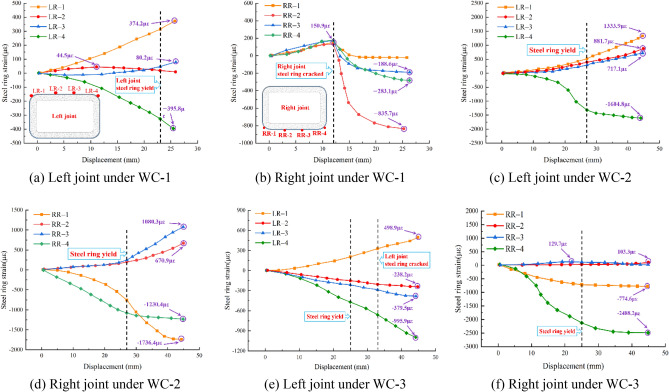
Steel ring strain.

Throughout the joint's resistance to shear deformation, the steel ring is the main force component. To explore the strain of the steel ring under varying foundation coefficients, Fig. 15 depicts the strain of the main stress parts of the steel ring under varying foundation conditions.

Under recent fill, the strain of the steel ring at measuring points LR-2 and LR-3 of the left joint is small, and the strain of the steel ring at measuring points LR-1 and LR-4 is large. All measuring locations on the steel ring of the right joint are in tension when the loading displacement is less than 12 mm. At the RR-1 measuring point, a crack appears when loaded to 12 mm. The strain at the RR-1 measuring point recovers to approximately 0 as the tension on the steel ring progressively relaxes. Eventually, the strain at the measuring point LR-1 and LR-4 at the chamfer of the left joint is the largest, which is 374.2 με and 395.8 με, respectively.

Under soft clay, the compressive strain at the LR-4 measuring point on the left joint rises faster with increasing loading displacement. This could be the result of improper welding of the steel ring or uneven assembly of the pipe sections. The tensile strain at measuring sites LR-1, LR-2, and LR-3 increases steadily with loading. At the right joint, measuring points RR-2 and RR-3 are in tension, whereas measuring points RR-1 and RR-4 are in compression at the chamfer. Eventually, the strains at the measuring points LR-1, LR-4, RR-1 and RR-4 at the chamfer of the two joints are the largest, which are 1333.9 με, 1604.8 με, 1736.4 με and 1230.4 με, respectively.

Under loose sand, the strain at the intermediate measuring points of the arch top and arch bottom of the steel ring on both the left and right joints is less, whereas the strain at the chamfer measuring points is greater. The strain rule of the steel ring correlates closely with the conclusion derived from measuring displacement with the pull wire displacement meter. This demonstrates that the arch top and arch bottom endure bending deformation towards the interior of the pipe, and a drum appears at the chamfer, indicating that the steel ring tends to flatten. Eventually, the strains at the measuring points LR-1, LR-4, RR-1 and RR-4 at the chamfer of the two joints are the largest, which are 498.9 με, 995.9 με, 774.6 με and 2488.2 με, respectively.

In conclusion, when a rectangular pipe jacking tunnel is subjected to shear load, the deformation of the arch top and arch bottom of the steel ring is relatively minor, whereas the drum phenomenon at the chamfer is the most severe, and cracking also occurs at the weld. The strain rate increases as the steel ring yields. After the steel ring cracks, its ability to withstand shear will diminish, and the strain rate will increase further. As the foundation coefficient decreases, the steel ring will enter the yield phase sooner. Therefore, the chamfer and weld of the steel ring require reinforcement to guarantee its normal operation.

### Concrete strain of the joints

**Figure 16:**
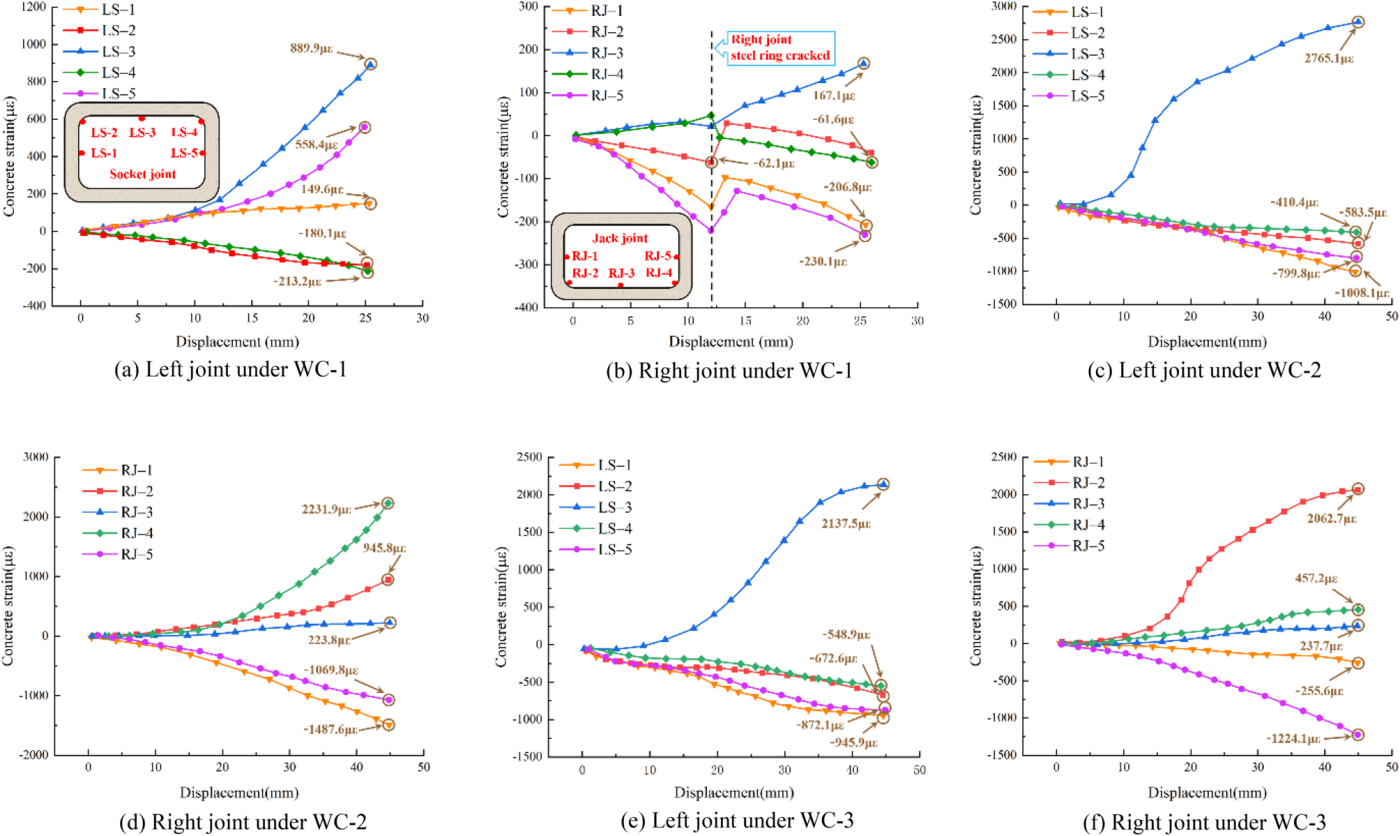
Concrete strain.

To investigate the strain of the joint's concrete under various foundation coefficients, Fig. 16 depicts the strain of the inner surface of the joint's concrete under varying working conditions.

Under recent fill, the tensile strain at the bottom of the socket joint is readily apparent, and the development law of compressive strain at the chamfer is similar. Due to the cracking of the steel ring at the location where the jack joint is loaded to 12 mm, the steel ring's restraining effect on the joint's concrete is weakened, and the strain of the concrete appears to recuperate temporarily. Eventually, the concrete strain at the top of the left joint is the largest, reaching 889.9 με.

Under soft clay, both sidewalls of the two joints are compressed, and the strain development follows a similar pattern. The compression of the chamfer at the socket joint and the relatively large tensile strain at the bottom indicate that the reaction force provided by the load-bearing structure to the pipe section is large. The strain at the bottom of the jack joint is comparatively small, whereas the tensile strain at the chamfer is extremely high, and the drum phenomenon of the steel ring is evident. Eventually, the concrete strain at the top of the left joint is the largest, reaching 2765.1 με.

Under loose sand, both sidewalls of the two joints are under compression, and the strain development trend is comparable. At the bottom of the joint, the tensile strain is relatively high, whereas the compressive strain at the chamfer is relatively low. The strain at the bottom of the jack joint is comparatively small, whereas the sidewalls are in a compression state and the chamfer is in a significant tension state. Eventually, the concrete strain at the top of the left joint is the largest, reaching 2137.5 με.

In conclusion, when shear failure occurs in a rectangular pipe jacking tunnel, the stress forms of the sidewalls at the socket joint and the jack joint are identical, whereas the stress forms of the bottom, the top, and the chamfer are distinct. First, both sidewalls of the two joints are in a state of compression deformation. Second, both the bottom and top of the two joints are compressed externally and pulled internally. The bottom is the result of the extrusion of the spring and load-bearing support, while the top is the result of the extrusion of the distribution beam and the steel ring. Finally, due to the various stress forms of the two joints, the chamfer of the socket joint is compressed, and the chamfer of the jack joint is tensioned. When the steel ring yields, the steel ring's limiting influence on the joint is diminished, and the strain rate of the concrete decreases. As the foundation coefficient decreases, the reaction force of the spring to the pipe section is reduced, and the final strain of the concrete decreases.

## Numerical analysis

This section uses ABAQUS finite element software to establish a rectangular pipe jacking model^23,24^ and analyse the shear response of the joint of the pipe section to better reveal the shear mechanical response of the F-type socket joint in a rectangular pipe jacking tunnel based on the laboratory joint test.

### Model and parameter configuration

**Figure 17:**
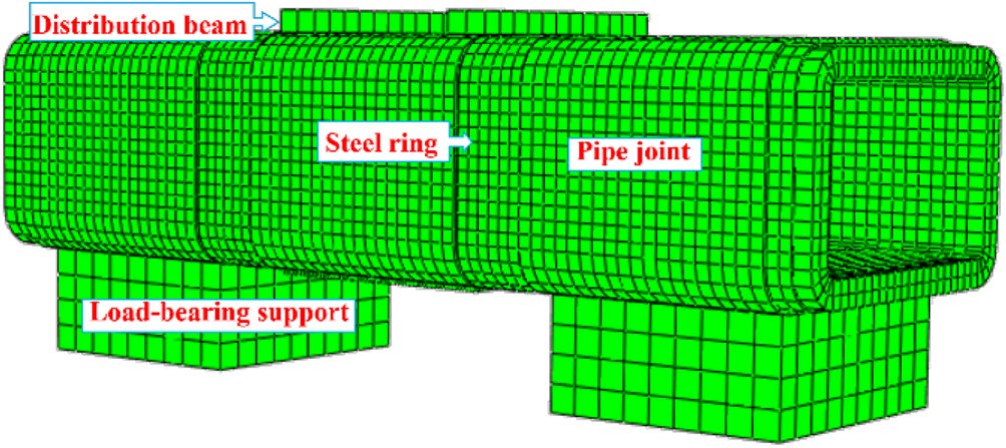
Finite element model.

As shown in Fig. 17, the model comprises a pipe section, steel ring, steel cage, distribution beam, and load-bearing support. The concrete, steel ring, distribution beam, and load-bearing support are simulated using entity units, while the steel bar adopts the truss element and is embedded in the concrete in an embedded mode, thereby improving the ability of the concrete to resist deformation. With the material parameters listed in Table 1, the concrete adopts a plastic damage constitutive model, the steel bar adopts an ideal elastic‒plastic constitutive model, and the steel ring adopts a double broken line constitutive model. By transmitting the load to the pipe section through the distribution beam, displacement loading is utilized to induce shear deformation in the pipe section.

The model's components are independent and require the establishment of contact relationships. The model employs a master–slave contact algorithm, with the binding constraint adopted between the steel ring and the socket joint's concrete, the pipe section and the distribution beam and the load-bearing support. Surface-to-surface contact is established between the steel ring and the jack joint's concrete, as well as between adjacent pipe sections. The tangent behaviour was set to penalty friction with a coefficient of friction of 0.2, and the normal behaviour was set to hard contact, allowing separation after contact.

### Results of the finite element simulation

**Figure 18:**
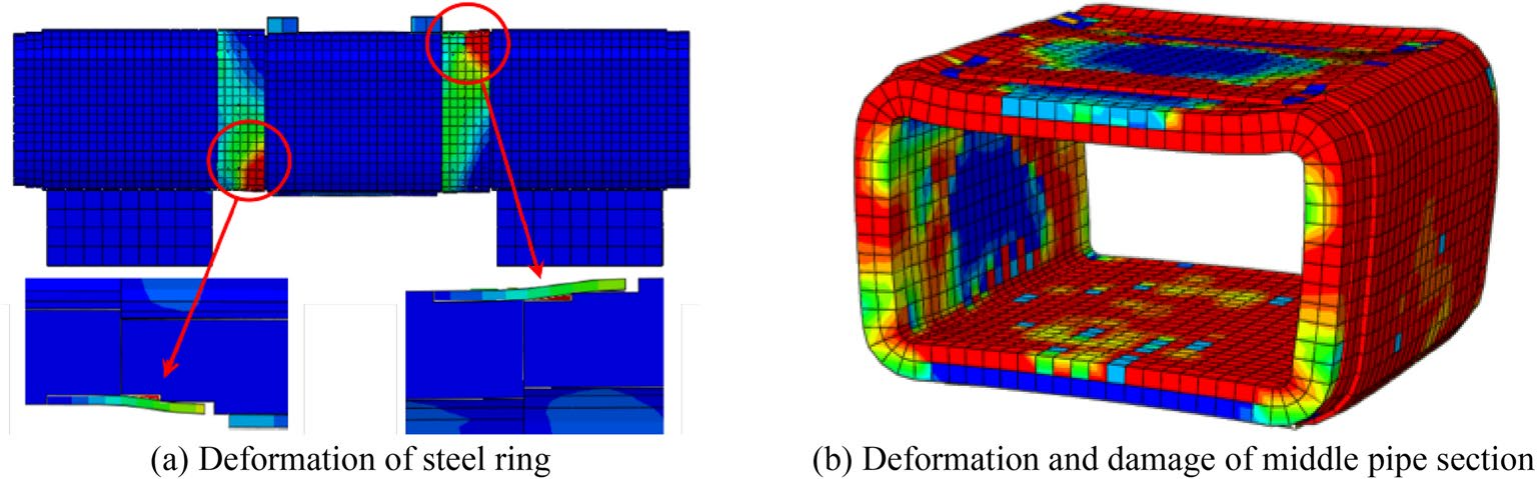
Modeling results.

**Figure 19:**
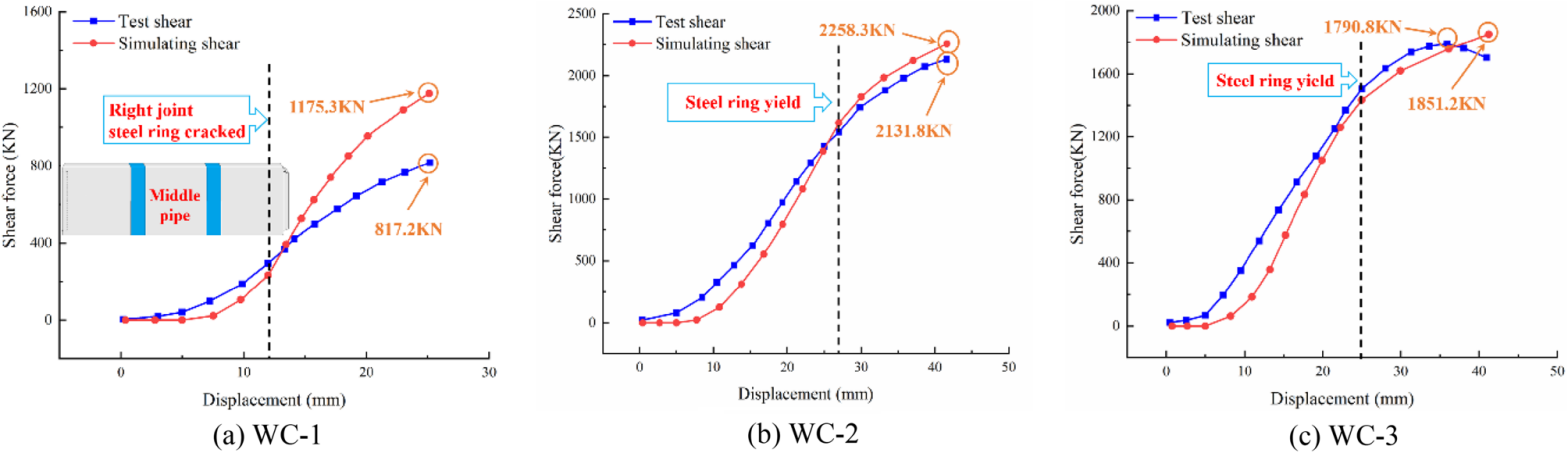
Comparative analysis of the joint shear.

Figure 18 depicts the calculation of the model. As the shear load increases, the chamfer of the steel ring is first in contact with the socket joint's concrete, and then the top and bottom of the pipe section gradually begin to sustain the load, exhibiting a centrosymmetric distribution of stress and deformation. Figure 19 compares the numerical simulation and experimental shear comparison curves of the middle pipe under various conditions. During the initial horizontal segment, no shear is generated, and the gap between the steel ring and the concrete is closed. The steel ring is in the elastic deformation stage in the second stage, which is characterized by a linearly increasing curve. The steel ring enters the yield stage in the third stage. It can be observed that the curve's growth rate decreases and the steel ring's shear capacity decreases. The development trend of the numerical simulation and experimental results is consistent.

### Deformation of the steel ring

**Figure 20:**
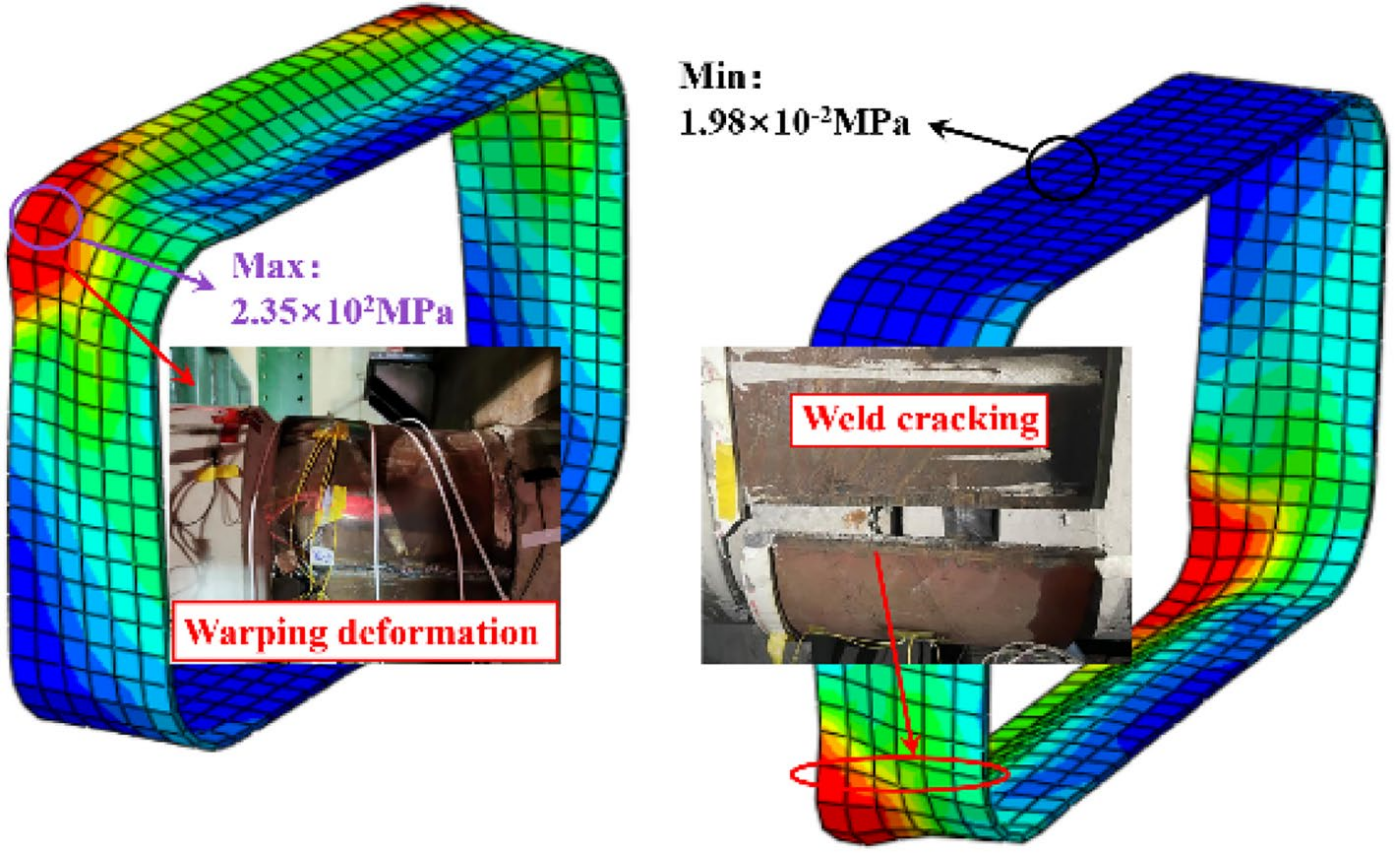
Stress distribution of the steel ring.

**Figure 21:**
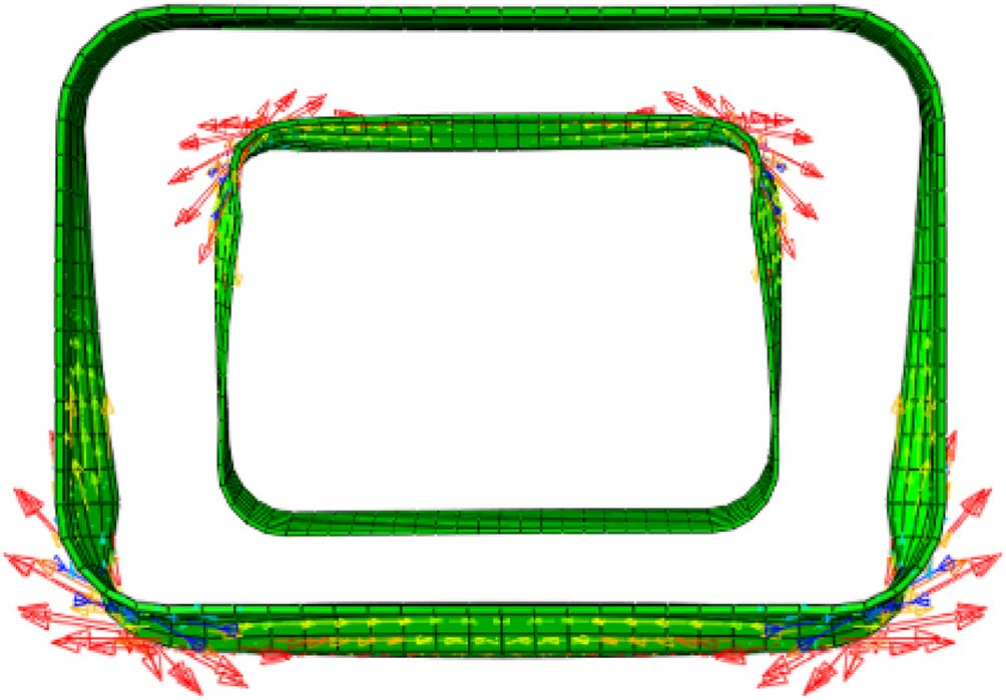
Stress vector distribution of the steel ring.

Figure 20 depicts the stress distribution of the steel ring during shear failure. As the load increases, the steel ring progressively yields and enters the plastic failure stage, and the failure surface expands and spreads continuously. The deformation of the steel ring on both sides is centrosymmetric, with the greatest deformation occurring at the chamfer and the least deformation occurring at the top and bottom of the arch. The chamfer yield failure area accounts for approximately 15% of the whole steel ring, which is consistent with the test results. As shown in Fig. 21, stress vector diagrams of the steel ring were extracted to further analyse the stress distribution inside the steel ring. The stress is primarily concentrated on the four chamfers, with a maximal value occurring in the centre of each chamfer and a gradual decrease on both sides. As a result, the stresses at the top, bottom, and sidewall of the steel ring are significantly less than those at the chamfers and are always in the elastic stage.

### Concrete damage

**Figure 22:**
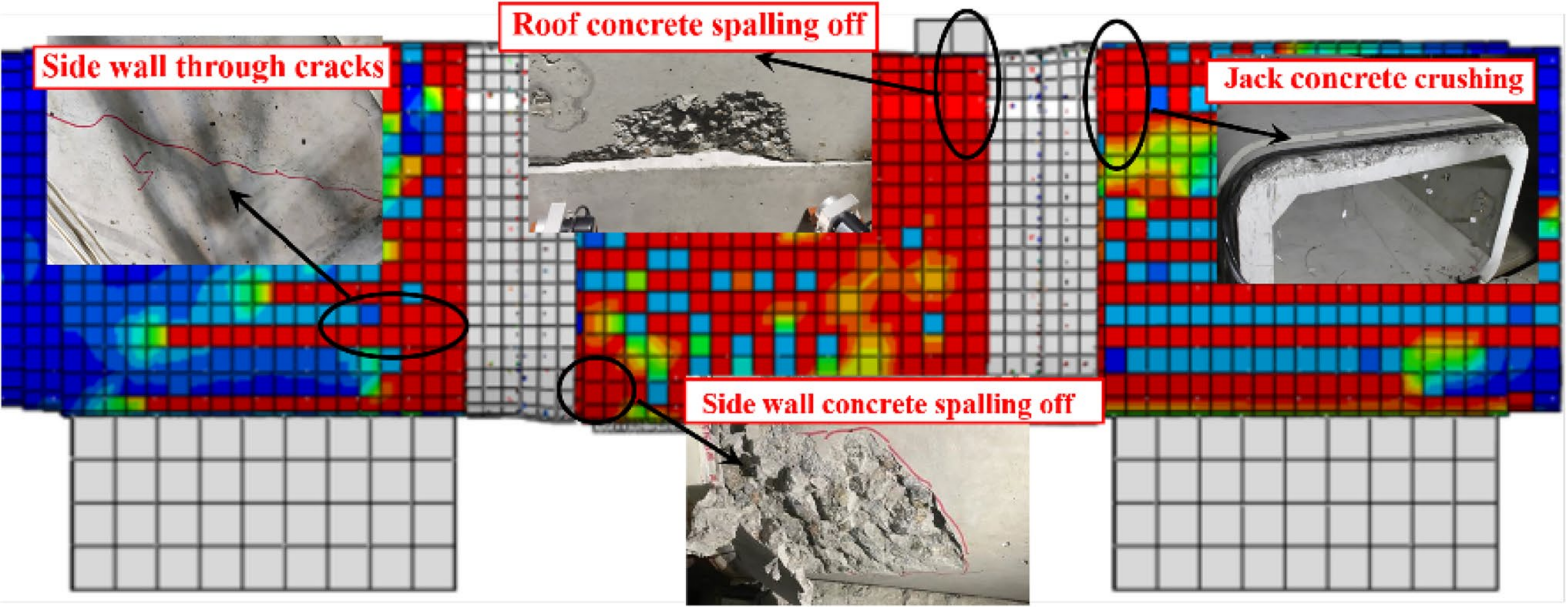
Damage of the pipe section.

Figure 22 depicts the damage state of the pipe section during shear failure. When the rectangular pipe jacking is subjected to shear load, the middle pipe section is in the extrusion state as a whole, and the compression damage range of the pipe section is larger than the tensile damage range. Therefore, the compression damage state of the pipe section is mainly analyzed. The concrete has a larger range of damage, with the damaged area comprising approximately 40% of the complete pipe section. The experimental failure phenomenon resembles the damage characteristics of the model. The concrete near the distribution beam fails first, with the upper load and the reaction force of the bottom springs continuously compressing the pipe section, causing the gradual formation of through cracks. The damage on the inner surface of the top is greater than that on the outer surface because the inner surface is under tension and the tensile strength of concrete is significantly less than its compressive strength. The jack joint's concrete is severely damaged due to the steel ring's compression of the concrete, and the damage extends continuously from the joint to the interior of the pipe. This is consistent with the experimental findings, which show that the cracks at the joint are continuously spreading alongside the phenomenon of concrete spalling off.

### Impact of various steel ring thicknesses

To examine the effect of the thickness of the steel ring on the shear capacity of the joint, numerical simulations were conducted using steel rings with thicknesses of 11 mm, 14 mm, and 17 mm based on the "Technical specification for pipe jacking engineering with rectangular cross section"^25^.

**Figure 23:**
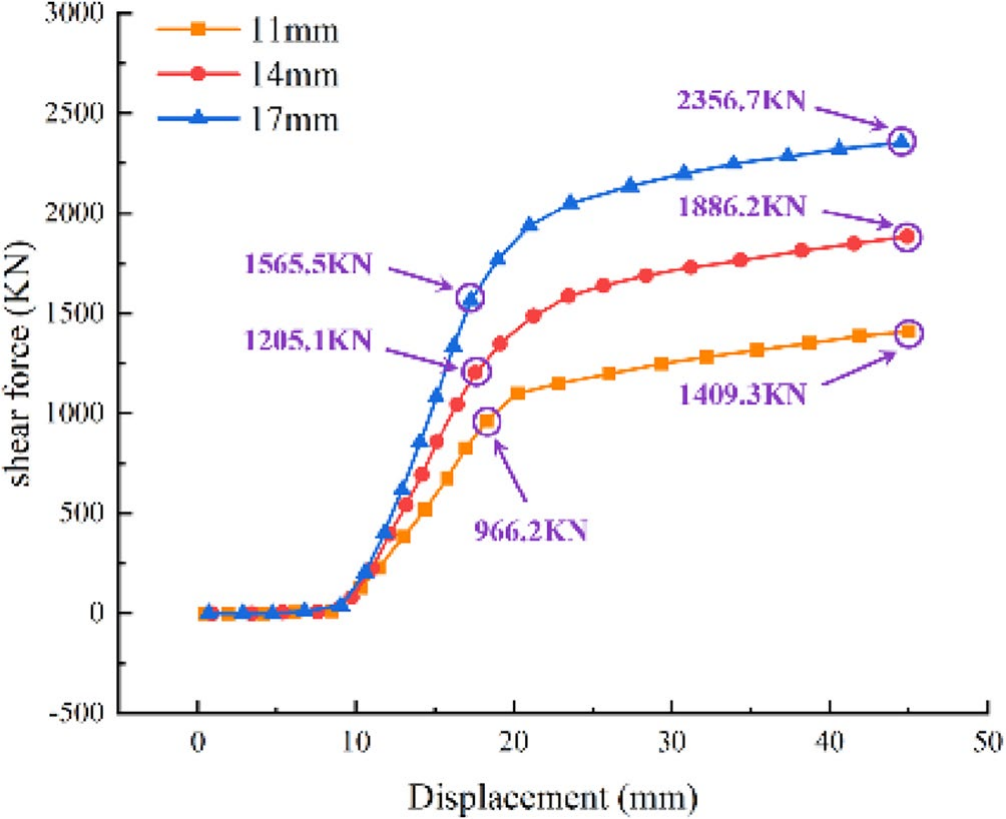
Shear‒displacement curves of various steel ring thicknesses.

As demonstrated in Fig. 23, the larger the thickness of the steel ring is, the greater its shear capacity. The variation trend between various thicknesses, which can be divided into the horizontal stage, elastic stage, and yield stage, is essentially identical, as are the growth rates of shear in the yield stage. Similarly, the shear capacity in the elastic stage increases by 25–30% for every 3 mm increase in the thickness of the steel ring, while the ultimate shear capacity increases by 25–34%. Therefore, when the thickness of the steel ring is in the range of 10–20 mm, the thickness of the steel ring can be appropriately increased to improve the bearing capacity of the joint.

### Joint shear process

According to the results of the experiments and numerical simulations, the overall shear capacity of the F-type socket joint in a rectangular pipe jacking tunnel varies in four stages:

During the initial stage, when the shear is comparatively low, the steel ring gradually tightens the rubber ring, and the various parts of the joint begin to come into contact, causing the 5 mm design clearance to gradually close. At this moment, the shear is less than the static friction force generated by the assembly pressure, and most of the shear is carried by the static friction force between the joint concrete. The joint does not endure significant dislocation at this stage.

As the shear progressively increases and surpasses the static friction force of the joint, the steel ring first contacts the jack joint's concrete. The steel ring then absorbs the increasing shear, resulting in shear dislocation at the joint. At this point, both the dislocation and the rate of deformation are relatively small. The steel ring's displacement and deformation are transmitted laterally.

Third, as the shear increases, the yield point appears first at the chamfer. Once the steel ring enters the yielding stage, the rate of deformation of the steel ring and shear dislocation in the joint accelerate. Consequently, the steel ring begins to drum and warp, and numerous cracks appear along the sidewall of the pipe section.

The joint finally enters the plastic stage. At this stage, the concrete in the joint is crushed and spalls off, and cracks near the joint gradually penetrate. The phenomenon of drum and warp of the chamfered steel ring becomes significant, and the joint eventually fails due to shear failure.

Combined with the joint test and numerical simulation, when the rectangular pipe jacking tunnel is subjected to shear load, the damage at the joint is the most serious, and the stress concentration will occur at the chamfer, which needs to be strengthened locally.

## Conclusions

The shear mechanical response and deformation failure of the joint are explored using a joint shear test of an F-type socket joint in a rectangular pipe jacking tunnel combined with numerical simulation, and the following findings are made:

1. When the foundation coefficient decreases by 33%, the bearing capacity of shear force at the joint decreases by about 12%. And with the weakening of the foundation coefficient, the steel ring will enter the yield stage earlier, but the damage of the joint concrete will be reduced. Therefore, the joint concrete should be strengthened in the stratum with large foundation coefficient, and the steel ring should be strengthened in the stratum with small foundation coefficient.
2. During shear failure, the middle pipe section displays centrosymmetric failure, and stress concentration at the chamfer is likely to occur. The yield area of the steel ring chamfer comprises approximately 15% of the entire steel ring, whereas the concrete damage area comprises approximately 40% of the entire pipe section. At the interface between the steel ring and jack joint, the concrete is crushed, and through cracks are obvious along the edge of the pipe section.
3. The joint of the middle pipe relies primarily on the steel ring at the socket joint and the concrete at the jack joint to withstand shear, but the steel ring fails before the concrete. Therefore, it is essential to increase the strength of the steel ring to guarantee the joint's shear capacity. For every 3 mm increase in the steel ring thickness, the shear capacity in the elastic stage of the joint increases by 25% to 30%, and the ultimate shear capacity increases by 25% to 34%.
4. The shear deformation of the F-type socket joint in a rectangular pipe jacking tunnel can be broken down into four stages: clearance closure, elastic growth, shear strengthening, and yield failure. The joint's final failure characteristics are drum and warp of the steel ring, cracking of the steel ring weld, crushing of the concrete of the joint.
5. In the process of rectangular pipe jacking tunneling, the chamfering position is most likely to be damaged, so the weld should not be located at the chamfering position. The steel ring at the chamfering position needs to be locally strengthened, and the reinforcement needs to be added at the chamfering and top and bottom to improve the bearing capacity of concrete.

## Data Availability

The datasets used or analysed during the current study available from the corresponding author on reasonable request.
